# The 'Holy Grail' of shoulder dislocations: a systematic review on traumatic bilateral luxatio erecta; is it in reality a once-in-a-lifetime experience for an orthopaedic surgeon?

**DOI:** 10.1007/s00402-023-05047-x

**Published:** 2023-09-30

**Authors:** Dimitrios Ntourantonis, Vasileios Mousafeiris, Konstantinos Pantazis, Ilias Iliopoulos, Angelos Kaspiris, Panagiotis Korovessis, Ioanna Lianou

**Affiliations:** 1 Emergency Department, University Hospital of Patras, Patras, Greece; 2 Department of Medicine, School of Health Sciences, University of Patras, Patras, Greece; 3 Department of Orthopaedics, General Hospital of Patras, Patras, Greece; 4 Department of Orthopaedics, Central Hospital of Karlstad, Karlstad, Sweden; 5 AIMIS Healthcare Group, Larnaca, Cyprus; 6 Laboratory of Molecular Pharmacology, Department of Pharmacy, School of Health Sciences, University of Patras, Patras, Greece; 7 Olympion Medical Center, Patras, Greece

**Keywords:** Luxatio Erecta, Inferior shoulder dislocation, Bilateral

## Abstract

**Introduction:**

Even though shoulder dislocation is thought to be the most common dislocation treated in the Emergency Department, inferior ones, known as Luxatio Erecta, comprise only 0.5% of them. Taking into consideration the rareness of unilateral Luxatio Erecta, bilateral cases should be even fewer.

The purpose of this paper is to identify the reported number of cases of Traumatic Bilateral Luxatio Erecta in the literature over the last 100 years and to summarize the mechanism of injury, the initial management, and the complications of these patients.

**Materials and methods:**

We performed a systematic review of the literature regarding Traumatic Bilateral Luxatio Erecta. All articles published until 31st of December 2022 in PubMed and Google Scholar databases were searched using the terms “luxatio erecta”, ‘inferior dislocation”, and “bilateral”.

**Results:**

Eighty-two articles were retrieved from PubMed and Google Scholar search. Forty-four of them were initially included in our review. Six additional articles meeting the inclusion criteria were found from cross-references.

**Conclusion:**

The presence of this injury is extremely rare with only 51 cases in the literature. The incidence of concomitant injuries and complications seems to be extremely high and neurological deficits were detected on 42.8% of patients with Bilateral Luxatio Erecta. To our knowledge, this is the first systematic review of the literature regarding Traumatic Bilateral Luxatio Erecta that includes articles not only in English, a fact that provides more reliability on the estimation of the real number of cases of this rare injury compared to any other review on this subject to date.

## Introduction

Historically, shoulder dislocation is considered the most common dislocation treated in the Emergency Department. However, inferior dislocations, known as Luxatio Erecta (LE), comprise only 0.5% of all shoulder dislocations, compared to anterior and posterior ones, which account to 95–97% and 2–4%, respectively [1, 2]. The first case of LE was described by Middledorpf and Scharm in 1859 [3], and was unilateral. In 2018, Nambiar et al. [4] in their systematic review, they reported 199 cases of LE, including only 29 bilateral ones. We believe that this numbers does not reflect the reality as at least seven cases [5, 6] of unilateral LE were omitted by the authors, while at least four, have been published since then [7]. Because of its uncommon occurrence, and despite their unique clinical presentation, LE is often misdiagnosed as anterior dislocation. Neurovascular injuries, including neurapraxia of the brachial plexus, ulnar or radial nerve, axillary artery injury, or upper limb deep vein thrombosis seem to be common after inferior shoulder dislocation. Around 29% of all patients with unilateral LE present with neurological deficits and 9% of them with vascular compromise [4].

According to the literature, the vast majority of patients with Traumatic Bilateral Luxatio Erecta (TBLE) face some kind of complication. It has been reported that at least 60% of bilateral cases present with neurovascular deficits [4]; furthermore, musculoskeletal injuries including disruption of the supraspinatus, infraspinatus, subscapularis, and pectoralis major and/or fractures of the clavicle, coracoid, acromion, inferior glenoid, and greater tuberosity of the humerus are common after this type of dislocation. When ongoing pain or instability symptoms are suspected after initial management, MRI (Magnetic Resonance Imaging) scanning should be performed to demonstrate rotator cuff or labral injury.

Taking into consideration the rareness of unilateral LE, bilateral cases should be even fewer, but which is the real number of reported TBLE cases to date, which are the main mechanisms of injury that caused this rare clinical entity, which was the initial management of these patients and finally, with regard to the rates of complications, do they exhibit a higher prevalence when compared to the unilateral cases? Since the first case of TBLE by Murard [8] in 1920, there is no any systematic review analyzing these parameters.

## Materials and methods

### Systematic review methodology

#### Literature search

We performed a systematic review of the literature regarding TBLE. All articles published until the 31st of December 2022 in PubMed and Google Scholar databases were searched using the terms “luxatio erecta”, ‘inferior dislocation”, and “bilateral”. The search strategy for PubMed was: ((bilateral [Title/Abstract]) AND (luxatio erecta[Title/Abstract])) OR (inferior dislocation[Title/Abstract]), in Google Scholar, the advanced search option was used. The term “bilateral” was used in the field [with all of the words] while “luxatio erecta” and “inferior dislocation” were typed in the field [with at least one of the words] (allintitle: "bilateral" "luxatio erecta" OR "inferior dislocation"). Our study has been registered with the International Prospective Register of Systematic Reviews (PROSPERO) with registration number CRD42023392289.

#### Selection criteria

No language barriers were applied during our initial search. Inclusion criteria: case reports and/or small series. Table 1 summarizes the methodology of the systematic review that was conducted according to PRISMA guidelines [9]. Zotero version 6.0.10 by Digital Scholar (https://digitalscholar.org/) was used for removing the duplicated records.

**Table 1:**
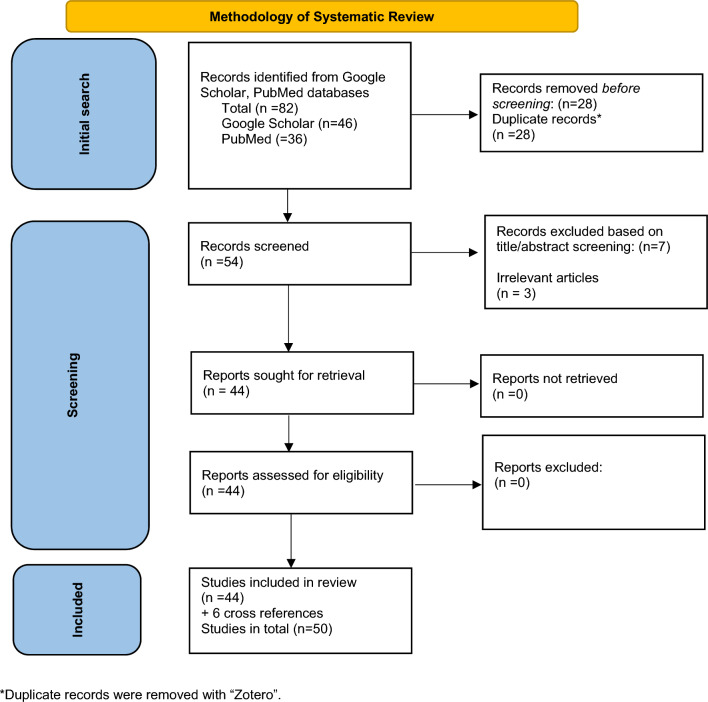
Methodology of systematic review according to PRISMA Guidelines

#### Data extraction

Data were extracted by standard form including patient demographics, mechanism of injury, reduction technique, and concomitant injuries and/or complications. Two authors (IL and VM) worked independently on screening the records. The selected articles were discussed for disagreements, and the senior author (DN) had the final decision for the inclusion of a study. After this stage, the two reviewers worked on the extraction of the data of interest. All results were collected and recorded in Microsoft Excel spreadsheet (Microsoft Office 365, Redmond, WA) by each reviewer independently. Finally, the reports were forwarded to the senior author. Any disagreement on the extracted data between the two reviewers for each individual study was resolved by the senior author by reviewing the articles of interest in detail. Difficulties were faced in the extraction of complications and/or concomitant injuries in the selected literature. After consensus, papers that clearly reported that there were no complications and/or concomitant injuries were signed as “no complications reported”. Papers with inadequate, or no information regarding this section were categorized as “not applicable” (N/A).

Descriptive statistics were performed using Microsoft Excel 365 (Microsoft Corporation, Redmond WA) to enable the data to be concise for presentation.

## Results

During our literature review, a total of 82 articles were revealed from PubMed and Google Scholar searches. Forty-four of them were initially included in our review. Furthermore, six additional articles meeting the inclusion criteria were found during the check of the reference lists of the retrieved articles (cross-references). After the initial screening of the articles, two tables were created. Table 2 includes all articles written or officially translated in English and Table 3 the ones written in different languages. This variation gave us the opportunity to estimate the real number of cases that have been published until now. For accuracy purposes of the details presented in this manuscript, only articles in English were used in the discussion section. Articles in different languages are presented separately (Table 3), providing details derived from English titles and/or abstracts when available. In total, 49 case reports and 1 small series [10] were included in our review.

**Table 2:** Summary of all reports originally published, or officially translated, in English

	First author, year		Sex			Age		MOI		Reduction technique		Concomitant injuries /complications
1	Peiro, 1975 [58]		M			49		Work equipment-related accident		Traction–counter-traction and axillary pressure to the humeral head		Lesion of left axillary nerve
2	Downey, 1982 [24]		M			57		Fall down a ladder		Not referred		N/A
3	Newman, 1993 [47]		F			75		RTA		Traction–counter-traction		Bilateral redislocation, left axillary nerve palsy (temporary)
4	Brady, 1995 [37]		F			80		Fall from standing height		Traction–counter-traction		No complication reported
5	Mesa, 1996 [48]		M			32		RTA		Traction–counter-traction		Sensory impairment of both median nerves, neurapraxia of left axillary nerve
6	Gelczer,1996 [35]		M			45		Horseback riding accident (fall)		Traction–counter-traction		Bilateral paresthesias of the ulnar, median, and radial nerves
7	Kumar, 2001 [38]		M			58		Fall from standing height		Traction–counter-traction		Bilateral brachial plexus injury and rotator cuff tears
8	Tsuchida, 2001 [36]		F			76		Fall from a boat while holding a tree		Traction–counter-traction		Right axillary nerve palsy
9	Mills, 2002 [27]		M			58		Fall down a ladder		Traction–counter-traction		No complication reported
10	Karaoglu, 2003 [28]		F			70		Fall down a ladder		Traction (general anesthesia)		Bilateral complete tear of the supraspinatus tendons
11	Sharma, 2005 [29]		F			75		Fall down a ladder		Traction–counter-traction		Left partial axillary nerve injury, complete rupture of left subscapularis and supraspinatus tendons
12	Elsayed, 2005 [19]		M			45		Fall from height		Traction–counter-traction		Bilateral axillary nerve palsy
13	Garcia, 2006 [49]		M			41		RTA		Traction–counter-traction		Left decreased C8 sensation, DVT of the left axillary and brachial vein
14	Sewecke, 2006 [50]		M			41		RTA		Traction–counter-traction		Left greater tuberosity fracture
15	Foad, 2007 [51]		M			40		RTA		Traction–counter-traction		Right greater tuberosity fracture*
16	Somville, 2008 [26]		M			45		Fall down a ladder		Closed, technique not referred (general anesthesia)		Left greater tuberosity fracture, subcapital (split head) fracture, left plexus injury Right greater tuberosity fracture
17	Musmeci, 2008 [39]		F			63		Fall from standing height		Traction–counter-traction		Right brachial plexus tear, Bilateral complete tear of the supraspinatus and infraspinatus tendons and a partial bilateral tear of the subscapularis tendon
18	Camarda, 2008 [30]		F			70		Fall down a ladder		Traction–counter-traction		Bilateral paresthesias of the arms (reversible)
19	Lee, 2009 [31]		F			57		Fall down a ladder		Closed, technique not referred		Right axillary nerve palsy
20	Groh, 2010 [10]		M			59		Fall down a ladder		Closed, technique not referred		Bilateral rotator cuff tear
21	Groh, 2010 [10]		M			40		Fall down a ladder		Closed, technique not referred		Axillary nerve injury without further details
22	Marks, 2011 [40]		F			59		Fall from standing height		Traction–counter-traction		Right greater tuberosity fracture
23	Acosta, 2012 [41]		M			43		Work related, fall from standing height		Traction–counter-traction		Right radial nerve palsy
24	Petty, 2013 [57]		M			68		Fall on a treadmill (Sport related injury)		Two-step technique		No complication reported
25	Saxena, 2013 [42]		M			19		Fall from standing height		Traction–counter-traction (general anesthesia)		Bilateral greater tuberosity fractures
26	Ellanti, 2013 [52]		M			19		RTA		Traction–counter-traction		Left paresthesia of C6, C7, Bilateral greater tuberosity fractures^**^
27	Crescibene, 2014 [55]		M			64		Trying to hold a heavy object above the head		Traction–counter-traction		No complication reported
28	Cacioppo, 2015 [20]		M			42		Fall from a height		Two-step technique		No complication reported
29	Fox, 2016 [21]		M			58		Fall from a height		Traction–counter-traction (right)/ two-step technique accidentally (left)		Bilateral decreased sensation of the axillary nerve, unilateral greater tuberosity fracture and labral tear^***^
30	Khedr, 2017 [22]		M			35		Fall from a height		Open reduction		Left greater tuberosity fracture
31	Reddy, 2018 [43]		M			36		Fall from standing height (seizure)		Traction–counter-traction		Bilateral greater tuberosity fractures
32	Ngam, 2018 [53]		F			59		RTA		Traction–counter-traction		Bilateral full-thickness supraspinatus tendon tears, left axillary neurapraxia
33	Lippert, 2018 [32]		M			70		Fall down a ladder		Traction–counter-traction		No complication reported
34	Biswas, 2019 [33]		M			66		Fall down a ladder		Traction–counter-traction (right)/ traction with anterior and downward pressure (left)		Left: massive full-thickness tear of the supraspinatus and infraspinatus, subscapularis torn Right: Hill–Sacks lesion, Bankart lesion, full-thickness tear of the supraspinatus and infraspinatus
35	Kessler, 2020 [44]		F			67		Fall from standing height		Traction and axillary pressure to the humeral head (right)/ two-step technique accidentally (left)		Right non-traumatic anterorinferior shoulder dislocation
36	Stirma, 2020 [34]		M			69		Fall down a ladder		Traction–counter-traction		Right: traumatic rupture of the supraspinatus Left: partial thickness tearing
37	Jayarajah, 2020 [23]		F			75		Fall from a height		Traction–counter-traction		No complication reported
38	Martinez-Romo, 2021 [54]		M			53		RTA (dragged by a vehicle)		Closed, technique not referred		N/A
39	Bawale, 2021 [25]		M			50		Fall down a ladder		Traction–counter-traction (general anesthesia)		No complication reported
40	Quesado, 2021 [45]		F			77		Fall from standing height		Traction–counter-traction		Left: internal proximal slope of the humerus Right: fracture of the anterior wall of the glenoid Bilateral partial rotator cuff tears
41	Ntourantonis, 2022 [56]		M			31		Trying to hold a heavy object above the head (sport-related injury)		Two-step technique		No complication reported
42	Güler, 2022 [46]		F			75		Fall from standing height		Traction–counter-traction		No complication reported

*M* male, *F* female, age in years, *MOI* mechanism of injury, *RTA* road traffic accident, *N/A* not applicable

*Polytrauma patient, bilateral knee dislocation

**Polytrauma patient, right tibial shaft fracture, bilateral pneumothoraxes

***Polytrauma patient, right open elbow fracture, T6 and L1 compression fractures

**Table 3:** Summary of all reports written in languages different than English

	First author, year	Age	Sex	Language	MOI	RT	Complications
1	Murard, 1920 [8]	N/A	N/A	German	N/A	N/A	N/A
2	Langfritz, 1956 [11]	N/A	N/A	German	N/A	N/A	N/A
3	Takamori, 1995 [14]	79	M	Japanese (abstract in English)	N/A	Traction–counter-traction	Right rotator cuff injury
4	Lill, 1996 [12]	36	M	German (abstract in English)	Fall from a height	Close reduction	N/A
5	Matehuala, 2006 [16]	58	M	Spanish (abstract in English)	N/A	N/A	N/A
6	Seo, 2009 [15]	N/A	M	Korean (abstract in English)	RTA	Close reduction	Bilateral greater tuberosity fracture
7	Milocevic, 2014 [17]	77	M	Bosnian (abstract in English)	Fall from a height	Traction–counter-traction	Bilateral brachial plexopathy, prominent on the right side
8	Madani, 2015 [18]	19	M	French	N/A	N/A	N/A
9	Völk, 2020 [13]	74	F	German	N/A	N/A	N/A

Age in years, *N/A* not applicable, *M* male, *F* female, *MOI* mechanism of injury, *RT* reduction technique, *RTA* road traffic accident

## Discussion

### Demographics

According to our literature review, TBLE seems to be an extremely rare injury, with only 51 cases to date in the literature. Forty-one papers (forty-two cases) were originally published in English and nine in other languages (German [8, 11–13], Japanese [14], Korean [15], Spanish [16], Bosnian [17], French [18]). Mean age of the patients in the English literature was 55 years old (range 19–80 years). There is a predominance of males compared to females with an incidence of 67% (28 out of 42 cases).

### Mechanism of injury

Falls were the commonest mechanism of injury in 32 out of 42 cases (76.2%), particularly high energy falls (fall from a height [19–23], ladder [10, 24–34] from a horse [35], and from a boat [36] accounting to 20 out of 42 cases (45,2%), while fall from standing height [37–46] accounting to 10 out of 42 cases (23,8%)). Road traffic accident was the cause of TBLE in eight cases [47–54] (19%). In two cases, the injury was related to an attempt to hold/prevent a heavy object from falling above the head [55, 56]. In one case, the patient fell from a docked boat while she was trying to grab a tree [36], while another patient sustained a fall against a wall [37]. Only two of the above-mentioned cases were related to a sports injury [56, 57]. No other information about the exact mechanism of fall were found in one report [53]. In the most uncommon mechanism of injury, patients’ arms were trapped in a cement mixer when the machine started accidentally [58], this mechanism of injury was categorized by the authors as a work-related one.

### Reduction techniques

Two main reduction techniques for LE have been described in the literature. The traction–counter-traction technique has been described by Freundlich et al. [59] and the two-step technique by Nho et al. [60] The second one involves a gentle rotation to change the position of the humeral head transforming the dislocation to an anterior one, and then on the second step, the reduction of the anterior shoulder dislocation with a maneuver of choice. These techniques may be performed with or without general anesthesia.

Close reduction has been achieved with traction–counter-traction in both sides in 30 cases [19, 23, 25, 27–30, 32–43, 45–53, 55, 58]. Different technique on each side was used in two cases [21, 44] and the two-step technique has been used in three cases [20, 56, 57]. General anesthesia was used in only four of the above-mentioned cases [25, 26, 28, 42]. Only one case required open reduction due to concomitant injuries [22].

Finally, in six cases, the reduction technique which has been used is not mentioned [10, 24, 26, 31, 54]

### Concomitant injuries—complications

TBLE is highly associated with neurovascular injuries. In our systematic review, out of 42 cases, neurological deficits were found in 18 [10, 19, 21, 26, 29–31, 35, 36, 38, 39, 41, 47–49, 52, 53, 58] (42,8%) including 1 case with bilateral complete brachial plexus tear [38] (accompanied with rotator cuff tear). On the other hand, vascular complications have been found in only one case. Particularly, Garcia et al. reported deep vein thrombosis extended from the left axillary to the proximal portion of the left brachial vein [49].

Proximal humeral fractures were reported in nine patients [21, 22, 26, 40, 42, 43, 50–52] (21,42%). Unilateral fracture of the greater tuberosity was found in five of these cases [21, 22, 40, 50, 51], while bilateral involvement was found in four patients [26, 42, 43, 52]. More severe fractures of the proximal humerus have been reported in only one case. In this case, a greater tuberosity fracture has been combined with a split head fracture [26].

Rotator cuff tears have been noted in nine patients [10, 28, 29, 33, 34, 38, 39, 45, 53] (21,42%), which were usually accompanied with concomitant injuries (mainly nerve injuries). The vast majority of the tears were bilateral [28, 29, 33, 34, 38, 39, 45, 53]. Labral tear has been reported in one case [21]. Redislocations were presented in three papers, including a case of bilateral redislocation after successful close reduction, which has been reported by Newman and Bendal [47], while Kessler et al. referred subsequent unilateral non-traumatic anterorinferior shoulder dislocation [44]. Somville [26] reported a case of an in-hospital humerus fracture and dislocation of the humerus head infraglenoidal in the left armpit after an initially successful reduction documented on a radiological basis.

No complications were reported in nine cases [20, 23, 25, 27, 32, 37, 46, 56, 57], while no further information was given in two papers [24, 54].

Summary of demographics, mechanism of injury, reduction technique, and complication rates of the cases published or officially translated in English is summarized in Table 4.

**Table 4:** Summary of demographics, mechanism of injury, reduction techniques and complications/concomitant injuries for papers written, or officially translated, in English

GENDER*	MOI*	RT**	CONCOMITANT INJURIES—COMPLICATIONS	(N)
MALE: 28 (67%)	Falls: 76,2%	TCT: 73,8%	**Neurological deficits**	**18**
FEMALE: 14 (33%)	HEF: 45,2%	N/A: 14,3%	**RC tears**	**9**
	FfSH: 31%	TST: 9,5%	-*Bilateral*	*8*
	RTA: 19%	OR: 2.4%	-*Unilateral*	*1*
	HOAH: 2.4%		**Fractures**	**12**
	WRI: 1,2		-*Unilateral GTF*	*5*
	N/A: 1,2		-*Bilateral GTF*	*4*
			-*Humeral neck fractures*	*1*
			-*Hill–Sachs lesion*	*1*
			-*Scapula fractures*	*1*
			**Redislocations**	**3**
			**Labral tears**	**1**
			**Vascular injuries**	**1**
			**No complications**	**9**
			**N/A**	**2**

Number of patients with complications/ concomitant injuries: 31/42 (73.8%) Number of shoulders with complications/ concomitant injuries: 56/84 (57.2%)

*MOI* mechanism of injury,* HEF* high energy fall,* FfSH* fall from standing height,* RTA* road traffic accident,* HOAH* holding object above head, * WRI* work-related injury,* RT* reduction technique,* TCT* traction–counter-traction,* TST* two-step technique,* OR* open reduction,* N/A* not applicable,* RC* rotator cuff,* GTF* greater tuberosity fracture

*Percentages were deducted based on a per-patient basis (n = 42) **percentages were deducted based on a per-injured shoulders basis (n = 84)

Based on the aforementioned findings, bilateral cases of LE are associated with a notably higher rate of complications compared to unilateral cases. Specifically, 73.8% of the patients (31 out of 42) with TBLE experienced some form of complication or concomitant injury (Tables 2, 4). When considering injured shoulders, the percentage of complications or concomitant injuries was also found to be substantial. Out of the 84 injured shoulders, 56 (57.2%) had some kind of complication or concomitant injury (Table 4). Furthermore, the incidence of soft tissue injuries like the ones on the rotator might be even higher, as in older published papers, there are no references regarding MRI results, a fact that could lead to the underestimation of this complication.

Nambiar et al. [4], in their systematic review regarding LE, reported a rate of neurological complications of 29%, while Gökkus et al. [61] estimated this incidence after LE to be up to 28%. Both studies included bilateral cases as well; particularly, Nambiar et al. encompassed 29 cases of TBLE, without providing further information regarding the analysis of papers with neurological complications. Conversely, Gökkus et al. presented a more comprehensive analysis, reporting 16 cases of LE with neurological deficits, of which 7 were bilateral. In both papers, the percentages were calculated on a per-patient basis. After analyzing these two systematic reviews, we hold a strong belief that incorporating bilateral cases does not offer a precise estimation of the rate of neurological complications. Our exclusive review, focusing solely on patients with TBLE, indicates that the rate of neurological deficits is approximately 42.8% on a per-patient basis (18 out of 42 cases). However, when considered on a per-shoulder basis, this percentage decreases to 28.57% (24 out of 84 injured shoulders) and remains consistent with the findings reported in the aforementioned analyzed papers regarding unilateral LE.

But which are the reasons behind the higher per-patient incidence of neurological complications in TBLE cases? The primary and clearly apparent answer for this question is the bilateral involvement itself. When both shoulders are dislocated, it mathematically doubles the potential for complications or concomitant injuries. Furthermore, to provide possible alternative explanations for this higher incidence of neurological complications on a per-patient basis, we propose a twofold hypothesis: first, the mechanism of injury in TBLE cases tends to be more severe compared to unilateral ones. The substantial forces involved in such injuries have the potential to inflict additional trauma upon the surrounding anatomical structures, including the nerves. Consequently, the likelihood of neurological complications is amplified, resulting to the observed higher incidence in TBLE cases. Second, the simultaneous dislocation of both shoulders in TBLE renders the upper trunk vulnerable and unprotected. This lack of safeguarding exposes critical neurovascular structures, such as the brachial plexus and the axillary nerve, to potential secondary damage. The compromised integrity of these vital components may contribute significantly to the increased occurrence of neurological complications.

While our hypothesis is based on the available evidence and logical reasoning, we acknowledge that further research is needed to validate and explore these factors in greater detail.

## Conclusion

To the best of our knowledge, this is the first systematic review of the literature solely focusing on TBLE. Additionally, the total case count includes articles published in languages other than English, enhancing the credibility of our estimation for the actual prevalence of this uncommon injury compared to all previous reviews on this subject to date.

But just how uncommon is TBLE in reality? In 1920, Murad [8], published the first documented case of TBLE, remarkably, even 103 years after this paper, the occurrence of this injury remains exceptionally rare. Up until December 31, 2022, only 51 cases have been reported in the literature. This translates to an incidence rate of 0.495 new cases per year since the initial publication. We strongly believe that the above-mentioned number of cases does not reflect the reality, as many simple and uncomplicated cases of TBLE have not been published. Senior author has treated six cases of TBLE since 2009, publishing only one due to its unique presentation [56]. ED physicians should maintain an increased awareness for prompt recognition taking into consideration the mechanism of injury, the distinctive signs and clinical presentation of this dislocation (hyperabducted and above the head-locked arm) and pitfalls in diagnosis and management must be avoided. The early recognition, followed by prompt, carefully performed reduction is crucial in avoiding soft tissue injuries (including vascular or brachial plexus injuries).

**Fig. 1:**
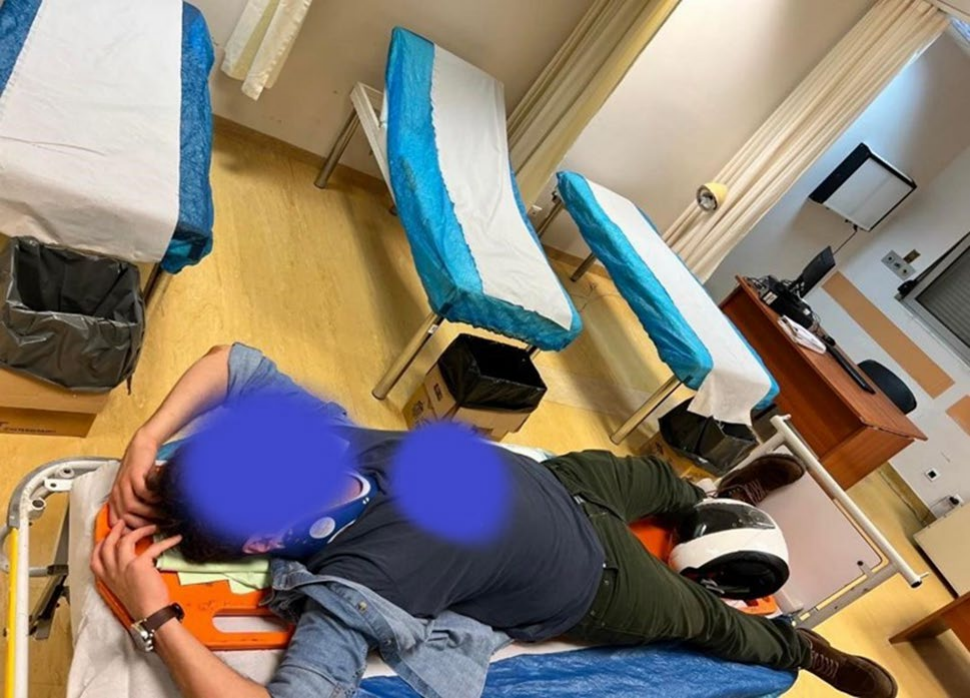
Characteristic TBLE patient’s position upon arrival at the Emergency Department

Great care should be given to polytrauma patients upon their arrival at the hospital. LE is relatively easy to diagnose given the unmistakable arm positioning (Fig. 1), but the presence of this injury increases the complexity of managing this type of patient in the ED due to the abducted arms.

### Limitations

In this article, we performed a systematic review of a rare clinical entity such as Traumatic Bilateral Luxatio Erecta. The results are based only on case reports that have been published and not on a general setting of large series, as the rareness of this traumatic pathology does not allow broader investigation settings. As a result, the quality of the reviewed article could not be documented.

The inclusion of only two databases in our review could be a limitation, but we strongly believe that the selected ones are ideal for the publication of this kind of reports and the number of cases, which are presented in this manuscript and the total number of cases is reflecting the reality.

## Data Availability

The data for this study is based on a systematic review of the existing literature on TBLE (Traumatic Bilateral Luxatio Erecta) as of December 31, 2022. All information and case data used in this research were obtained from publicly available academic and medical sources, including published articles and medical databases. No specific original data collection or experimentation was conducted for this study. Researchers interested in accessing the source materials and data used in this review should refer to the respective cited references and available academic sources.

## References

[1] Szalay E, Rockwood CJ (1984). Injuries of the shoulder and arm. Emerg Med Clin North Am.

[2] McLaughlin H (1952). Posterior dislocation of the shoulder. J Bone Jt Surg.

[3] Middledorff M (1859). De nova Humeri Luxationis specie. Clin Eur.

[4] Nambiar M, Owen D, Moore P, Carr A, Thomas M (2018). Traumatic inferior shoulder dislocation: a review of management and outcome. Eur J Trauma Emerg Surg.

[5] Matsumoto K, Ohara A, Yamanaka K, Takigami I, Naganawa T (2005). Luxatio erecta (inferior dislocation of the shoulder): a report of two cases and a review of the literature. Inj Extra.

[6] Yamamoto T, Yoshiya S, Kurosaka M, Nagira K, Nabeshima Y (2003). Luxatio erecta (inferior dislocation of the shoulder): a report of 5 cases and a review of the literature. Am J Orthop (Belle Mead NJ).

[7] Diallo M, Kassé AN, Mohamed Limam S, Sané JC, Dembélé B, Sy MH (2019). Erecta dislocation of the shoulder joint—A rare injury: About four cases. Clin Case Rep.

[8] Murard J (1920). Un cas de luxatio erecta de l’epaule, double et symetrique. Rev Orthop.

[9] Page MJ, Mckenzie JE, Bossuyt PM, Boutron I, Hoffmann TC, Mulrow CD (2020). The PRISMA statement: an updated guideline for reporting systematic reviews. BMJ.

[10] Groh GI, Wirth MA, Rockwood CA (2010). Results of treatment of luxatio erecta (inferior shoulder dislocation). J Shoulder Elb Surg.

[11] Langfritz HU (1956). Die doppelseitige traumatische Luxatio humeri erecta, eine seltene Verletzungsform [Bilateral traumatic luxatio hemeri erecta, an infrequent injury]. Monatsschr Unfallheilkd Versicherungsmed.

[12] Lill H, Sangmeister M, Hillrichs B, Lange K, Echtermeyer V. Die bilaterale Luxatio erecta des Schultergelenks - Eine seltene Verletzung. Management und Therapie bei einem polytraumatisierten Patienten. Unfallchirurg 1996;99:801–5. 10.1007/s001130050058. 10.1007/s001130050058 9005570

[13] Völk D, Crönlein M, Müller M, Biberthaler P, Kirchhoff C, Wurm M (2020). Luxatio erecta in bilateral traumatic shoulder dislocation. Unfallchirurg.

[14] Takamori H, Imasato H (1995). Bilateral Luxatio Erecta Orthop Traumatol.

[15] Seo J-B, Min B-K (2009). Bilateral Inferior Shoulder Dislocation with Greater Tuberosity Fracture - A Case Report -. J Korean Orthop Assoc.

[16] Matehuala García J, Peñafort García JA (2006). Luxación glenohumeral erecta bilateral. Acta Ortopéddica Mex.

[17] Milošević I, Božilović D, Jović V (2014). OBOSTRANA DONJA LUKSACIJA RAMENOG ZGLOBA (LUXATIO ERECTA HUMERI BILATERALIS) – PRIKAZ SLUČAJA. Timočki Med Glas.

[18] Madani T, Hani R, Karabila MA, Kharmaz M, El Ouadghiri M, Lahlou A (2015). La luxation erecta bilatérale: à propos d’un cas. Pan Afr Med J.

[19] Elsayed S, Hussein A, Konyves A, Jones DG (2005). Bilateral luxatio erecta humeri Inj Extra.

[20] Cacioppo E, Waymack JR (2015). Bilateral inferior shoulder dislocation. West J Emerg Med.

[21] Fox AC, Martin DR (2016). Up in Arms: Bilateral Luxatio Erecta Fracture-Dislocations. Am J Orthop (Belle Mead NJ).

[22] Khedr H, Al-Zahrani A, Al-Zahrani A, Al-Qattan MM (2017). Bilateral irreducible inferior shoulder dislocation: A case report. Int J Surg Case Rep.

[23] Jayarajah U, Palkumbura C, Arulanantham A, Faleel A (2020). Unusual presentation of bilateral inferior shoulder dislocation following a trivial fall in an elderly female: a case report. J Gerontol Geriatr.

[24] Downey EF, Curtis DJ, Brower AC (1983). Unusual Dislocations of the Shoulder. Am J Roentgenol.

[25] Bawale R, Soliman A, Jain R, Singh B (2021). Bilateral Luxatio Erecta Etiology Diagnosis and Management. Open Sci J..

[26] Somville FJMP (2016). Plexus Injury after Reduction of Anterior Caudal Dislocation of the Shoulder. Acta Chir Belg.

[27] Mills LD, Barrows T, Benitez F (2003). Bilateral luxatio erecta. J Emerg Med.

[28] Karaoglu S, Guney A, Ozturk M, Kekec Z (2003). Bilateral luxatio erecta humeri. Arch Orthop Trauma Surg.

[29] Sharma H, Lindsay JR (2005). An unusual presentation in the emergency department with “hands up” posture. Hosp Med.

[30] Camarda L, Martorana U, D’Arienzo M (2009). A case of bilateral luxatio erecta. J Orthop Traumatol.

[31] Lee AJ, Hardy PJ, Kitchen E, Shahane S (2009). Luxatio erecta: A prehospital challenge in patient packaging. Emerg Med J.

[32] Lippert J, Desai B (2018). Bilateral Luxatio Erecta: A Case Report. Case Rep Clin Med.

[33] Biswas S, Peirish R (2019). Traumatic Bilateral Luxatio Erecta from a Sliding Injury Down a Ladder; A Rare Case Report and Literature Review. Bull Emerg Trauma.

[34] Stirma GA, Secundino AR, Baracho FR, Dau L (2020). Bilateral Erecta Luxation: A Case Report and Literature Review. JBJS Case Connect.

[35] Gelczer RK, Swee RG, Adkins MC (1996). Bilateral inferior glenohumeral dislocations. J Trauma-Inj Infect Crit Care.

[36] Tsuchida T, Yang K, Kimura Y, Taniwaki M, Ishigaki S, Itoi E (2001). Luxatio erecta of bilateral shoulders. J Shoulder Elb Surg.

[37] Brady WJ, Knuth CJ, Pirrallo RG (1995). Bilateral inferior glenohumeral dislocation: Luxatio erecta, an unusual presentation of a rare disorder. J Emerg Med.

[38] Kumar KS, O’Rourke S, Pillay JG (2001). Hands up: a case of bilateral inferior shoulder dislocation. Emerg Med J.

[39] Musmeci E, Gaspari D, Sandri A, Regis D, Bartolozzi P (2008). Bilateral Luxatio Erecta Humeri Associated With a Unilateral Brachial Plexus and Bilateral Rotator Cuff Injuries: A Case Report. J Orthop Trauma.

[40] Marks TOMC, Kelsall NKR, Southgate JJ (2011). Bilateral luxatio erecta: Recognition and reduction. EMA - Emerg Med Australas.

[41] Acosta CAX, da Silva Resch E, Rodrigues R (2012). Bilateral Luxatio Erecta, a case report. Rev Bras Ortop (English Ed).

[42] Saxena V, Pradhan P (2013). Bilateral luxatio erecta with greater tuberosity fracture: A case report. J Clin Orthop Trauma.

[43] Reddy SV, Jaiswal A, Kanwar CS (2019). A rare case of bilateral luxatio erecta with bilateral greater tuberosity fracture following a fall due to seizure. J Clin Orthop Trauma.

[44] Kessler A, Hinkley J, Houserman D, Lytle J, Sorscher M (2019). Bilateral Luxatio Erecta Humeri With Acute Anterior-inferior Re-dislocation. Clin Pract Cases Emerg Med.

[45] Quesado M, Soares D, Afonso J, Lopes D, Silva F, Mendes J (2021). Bilateral Luxatio Erecta: An Atypical Presentation at the Emergency Department. Case Rep Orthop Res.

[46] Güler S, Kocaşaban DÜ. Bilateral Luxatio Erecta. Ajmhs 2022;58.

[47] Newman KJH, Bendall R (1993). Bilateral inferior shoulder dislocation: both subglenoid and subcoracoid types seen in the same patient. Injury.

[48] Mesa M, Carpintero P, Carpintero J (1996). Bilateral luxatio erecta humeri. Acta Orthop Belg.

[49] Garcia R, Ponsky T, Brody F, Long J (2006). Bilateral luxatio erecta complicated by venous thrombosis. J Trauma.

[50] Sewecke JJ, Varitimidis SE (2006). Bilateral luxatio erecta: a case report and review of the literature. Am J Orthop (Belle Mead NJ).

[51] Foad A, LaPrade RF (2007). Bilateral Luxatio Erecta Humeri and Bilateral Knee Dislocations in the Same Patient. Am J Orthop.

[52] Ellanti P, Davarinos N, Connolly MJ, Khan HA (2013). Bilateral luxatio erecta humeri with a unilateral brachial plexus injury. J Emergencies, Trauma Shock.

[53] Ngam PI, Hallinan JT, Sia DSY (2019). Sequelae of bilateral luxatio erecta in the acute post-reduction period demonstrated by MRI: a case report and literature review. Skeletal Radiol.

[54] Martinez-Romo M, Lotfipour S, McCoy C (2021). Bilateral luxatio erecta humeri. Clin Pr Cases Emerg Med.

[55] Crescibene A, Sbano R, Martire F, Candela M (2014). Bilateral inferior dislocation of the shoulder joint. Minerva Ortop e Traumatol.

[56] Ntourantonis D, Lianou I, Ampariotou A, Daskalopoulos V (2022). “Hands Up” and Social Distancing: A Rare Case of Bilateral Luxatio Erecta During the Second Wave of the COVID-19 Pandemic Lockdown Period. Cureus.

[57] Petty K, Price J, Kharasch M, Novack J (2014). Bilateral luxatio erecta: A case report. J Emerg Med.

[58] Peiró A, Ferrandis R, Correa F (1975). Bilateral erect dislocation of the shoulders. Injury.

[59] Freundlich BD (1983). Luxatio erecta. J Trauma.

[60] Nho SJ, Dodson CC, Bardzik KF, Brophy RH, Domb BG, MacGillivray JD (2006). The two-step maneuver for closed reduction of inferior glenohumeral dislocation (luxatio erecta to anterior dislocation to reduction). J Orthop Trauma.

[61] Gökkus K, Sagtas E, Saylik M, Aydin AT, Atmaca H (2015). Luxatio erecta humeri: Report of a swimming injury with analysis of the mechanism of the injury and associated injuries in literature. J Emerg Trauma Shock.

